# Three-Dimensional Heads-up Cataract Surgery Using Femtosecond Laser: Efficiency, Efficacy, Safety, and Medical Education—A Randomized Clinical Trial

**DOI:** 10.1167/tvst.10.9.4

**Published:** 2021-08-03

**Authors:** Kai Wang, Fan Song, Lifang Zhang, Jia Xu, Yueyang Zhong, Bing Lu, Ke Yao

**Affiliations:** 1 Eye Center of the Second Affiliated Hospital, School of Medicine, Zhejiang University, Hangzhou, Zhejiang Province, China

**Keywords:** heads-up cataract surgery, 3D visualization system, efficiency, efficacy, safety, medical education

## Abstract

**Purpose:**

To compare the efficiency, efficacy, and safety, as well as the educational value, of heads-up (three-dimensional visualization system–assisted) and traditional microscopic cataract surgery.

**Methods:**

This randomized noninferiority trial enrolled 242 eyes of 201 patients who received femtosecond laser-assisted cataract surgery. The questionnaire study enrolled 26 medical interns and 39 medical students. Patients received surgery under either a three-dimensional visualization system (3D group, 117 eyes) or traditional microscope (TM group, 125 eyes) after random allocation. The primary outcome was surgical time. The noninferiority margin of surgical time was 60 seconds. Secondary outcomes included ultrasound power, phacoemulsification time, visual acuity, intraocular pressure, endothelial cell density, central corneal thickness, complications, and observer satisfaction scores for surgical procedures.

**Results:**

Surgical time was 462.03 ± 80.36 seconds in the 3D group and 452.13 ± 76.63 seconds in the TM group (difference 9.90 seconds; 95% CI, –9.98 to 29.78; *P* = 0.365). Visual acuity and other perioperative parameters were comparable between the 3D group and the TM group (all *P* > 0.05). Incidences of both intraoperative and postoperative complications were low and not statistically different between groups (all *P* > 0.05). Across all observers, 3D surgery was superior to TM surgery for improving the degree of satisfaction (all *P* < 0.001).

**Conclusions:**

The surgical efficiency of heads-up cataract surgery is not inferior to traditional microscopic surgery. Both methods achieved similar efficacy and safety outcomes. Moreover, heads-up cataract surgery showed a significant advantage in medical education.

**Translational Relevance:**

Our findings show that heads-up cataract surgery has comparable efficiency, efficacy, and safety, as well as superior medical educational value, to TM surgery, which lays the foundation for promoting and popularizing this technology.

## Introduction

Cataract extraction with intraocular lens (IOL) implantation is the most established method of cataract management so far. However, conducting surgery through a microscope might result in an uncomfortable body posture with chronic cervical and lumbar fatigue for some surgeons, causing cumulative work-related musculoskeletal disorders that may shorten their career.^1^

Unlike traditional microscopic (TM) surgery, heads-up cataract surgery involves two high-definition cameras that capture image signals from different angles of view under the microscope. These are then processed by an image processor and transmitted to a high-resolution three-dimensional (3D) screen. Surgeons wearing passive polarized 3D glasses perform microsurgical procedures by directly viewing screen; they do not have to look down through the microscope eyepieces during the surgery.

Heads-up surgery has the advantage of high-resolution visualization, superior stereoscopic sensation, and wide visual field.^2^ Its appropriate ergonomic design enables a more natural body posture and reduces the burden on surgeons’ cervical spines.^2^^–^^5^ Moreover, 3D imaging allows observers to see exactly what the surgeon sees during the surgery, improving effectiveness in teaching and learning.^4^^,^^6^^,^^7^

Since first developed in 2009,^8^ heads-up surgery has gained increasing popularity in ophthalmic surgery, including Descemet membrane endothelial keratoplasty (DMEK),^9^^,^^10^ cataract surgery,^11^^–^^14^ and vitreoretinal surgery.^4^^,^^15^^–^^19^ Current reports on heads-up surgery mainly focus on its use in vitreoretinal disease because of its advantage of a lower endoillumination level and higher depth of field.^4^^,^^15^^–^^19^ However, as manipulation space is smaller in the anterior segment compared with the posterior segment, heads-up cataract surgery is performed less frequently than TM surgery.

A retrospective study reported the efficiency and safety of heads-up surgery on aspects of surgical duration and complications.^14^ However, to date, no prospective randomized trial with a large sample has comprehensively evaluated this technology.

The present study evaluated the efficiency, efficacy, and safety of heads-up cataract surgery compared with TM surgery, as well as its educational value in intraoperative surgical procedures.

## Methods

### Study Design and Approvals

This randomized clinical trial was conducted from February 10, 2020, to June 12, 2020, at the Second Affiliated Hospital of Zhejiang University School of Medicine, Hangzhou, China, after obtaining approval from the institutional review board and in accordance with the Declaration of Helsinki. It was registered with the Chinese Clinical Trial Registry (ChiCTR2000029466) in February 2, 2020. Written informed consent was obtained from all patients after full explanation of the study.

### Patients

This study consecutively recruited Han Chinese patients with age-related cataracts who underwent femtosecond laser-assisted cataract surgery with a posterior chamber IOL insertion. Exclusion criteria comprised (1) lack of cooperation; (2) coexisting macular pathologies, ocular surface abnormalities, or serious ocular disorders, including uncontrolled glaucoma, high myopia, retinal detachment, and dry eye syndrome; (3) poorly dilated pupils or iris abnormality; (4) ocular trauma, surgery, or inflammation history; (5) rheumatic diseases, diabetes, or any other systemic diseases history; (6) current or recent use of steroids; and (7) known sensitivity to concomitant medications used perioperatively.^20^ When both eyes of one patient were eligible, both eyes were included in the study.

### Randomization

**Figure 1.**
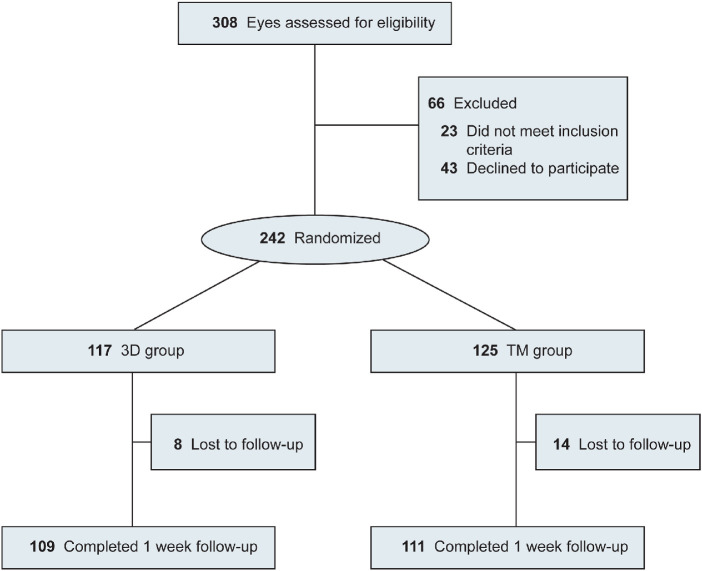
Study design flow diagram. 3D group: the group that received heads-up cataract surgery; TM group: the group that received traditional microscopic cataract surgery.

Eligible eyes were randomized into two groups to undergo cataract surgery using either a heads-up 3D-display system (3D group) or a traditional binocular microscope (TM group) (Fig. 1). Computer-generated tables (SPSS software version 24.0; SPSS, Inc., Chicago, IL, USA) with an allocation ratio of 1:1 were used.

### Surgical Technique

**Figure 2.**
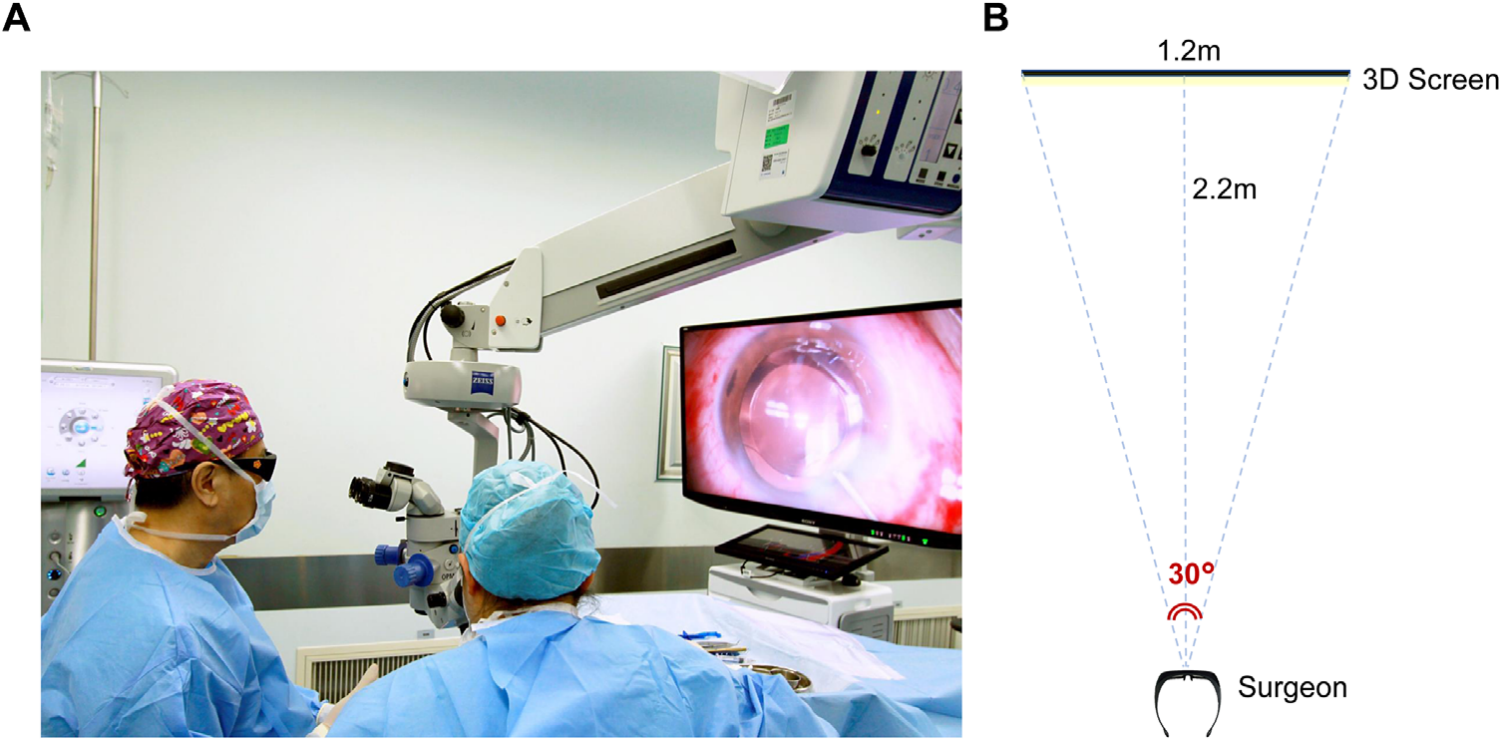
Intraoperative images of heads-up cataract surgery using 3D visualization system. (A) The surgeon wearing passive polarized 3D glasses is performing surgery by looking at the high-definition 3D screen, which is 2.2 m away from him. (B) Sketch map of the setup of the 3D screen.

The same experienced surgeon (KY) performed all surgical procedures using topical anesthesia, standard femtosecond laser platform (LenSx version 2.23; Alcon-LenSx, Aliso Viejo, CA, USA), and a phacoemulsification system (Stellaris system; Bausch & Lomb, New York, NY, USA). After the femtosecond laser pretreatment, the surgery was performed with a traditional binocular microscope for the TM group. For the 3D group, surgery was performed with a 3D visualization system (NCVideo3D system; NewComm, Beijing, China) comprising a 55-inch LCD monitor with a 4K display (LMD-X550TC; Sony, Tokyo, Japan), as shown in Figure 2. The system delay was 60 ms. Both the heads-up path and microscope eyepiece were available for observation and could be switched as needed. More than 200 cases of heads-up cataract surgery were performed by the surgeon prior to the initiation of the present study.

All patients were prescribed topical levofloxacin 0.5% four times for 1 day before surgeries. Pupillary dilation was achieved with the instillation of one drop of tropicamide every 15 minutes for three times. All patients underwent femtosecond laser capsulotomy with a diameter of 5.0 mm and nuclear fragmentation. After that, a 0.8-mm side-port incision and a 2.0-mm primary single-plane clear corneal incision were made manually using a keratome. The anterior capsule was removed with capsule forceps, followed by phacoemulsification using a standard stop-and-chop technique and insertion of an IOL. All procedural characteristics of the phacoemulsification were consistent between the two groups. After surgeries, topical dexamethasone tobramycin four times a day for 2 weeks and pranoprofen four times a day for 1 month were given to all patients.^21^

### Outcome Measures

The primary outcome was surgical time (initiated by side-port corneal incision and finished with hydration of primary corneal incision). The secondary outcomes were other perioperative clinical parameters.

All eligible patients were interviewed regarding their medical histories and underwent a comprehensive ophthalmic examination, including uncorrected distance visual acuity (UDVA), slit-lamp biomicroscopy, dilated fundoscopy, intraocular pressure (IOP) measurement (NT-510; Nidek, Gamagori, Japan), and corneal topography by Scheimpflug imaging (Pentacam; Oculus Optikgeräte GmbH, Wetzlar, Germany). The nuclei were graded according to the Emery–Little classification.

The evaluated intraoperative parameters included the surgical time, ultrasound (US) power, absolute phacoemulsification time (APT), and effective phacoemulsification time (EPT). Intraoperative complications—including posterior capsule rupture with or without vitreous loss, intraoperative iris floppy syndrome or iris prolapse, iris injury, lens materials dropped into vitreous and suprachoroidal effusion with or without hemorrhage— were recorded.

UDVA, IOP, endothelial cell density (ECD), and central corneal thickness (CCT) were measured at 1 day and 1 week postoperation. UDVA measured with the Snellen visual acuity chart was converted to the logarithm of the minimum angle of resolution equivalent for statistical analysis. ECD was measured with a noncontact autofocus EM-3000 specular microscope (Tomey, Nagoya, Japan). Endothelial cell loss (ECL) was calculated as a percentage of the preoperative ECD. CCT was measured using Scheimpflug imaging.

All measurements were conducted by the same technician, who was masked to the patients, and the conditions were kept consistent for all operated eyes. Postoperative complications were recorded, including IOP spike (defined as IOP ≥25 mm Hg or an elevation of IOP ≥8 mm Hg from the baseline IOP^22^), corneal edema, toxic anterior segment syndrome, intraocular lens decentration and dislocation, retained lens materials, wound leak or rupture, hyphema, and endophthalmitis.

### Questionnaire Evaluation

Our questionnaire was adapted from a preexisting questionnaire found in the literature review of similar studies.^4^^,^^7^ At the end of each surgical session, all observers (including medical interns and medical students) were asked to rate their satisfaction on a scale of 1 to 10, representing low to excellent, for both types of surgery. The five items they were asked to rate were depth of field, visibility, detail understanding (including the incision, phacoemulsification, irrigation/aspiration, lens insertion/rotation, and wound closure, etc.), knowledge retention (including the surgical procedure, notice, etc.), and educational value (including the technical knowledge and detail acquisition, communication, improving intraocular spatial awareness, decision-making instruction, etc.).

### Statistical Analysis

The trial was framed as a noninferiority design to demonstrate that surgical time of heads-up cataract surgery is not inferior to that of traditional microscopic cataract surgery. We set the noninferiority margin of surgical time for the primary outcome at 60 seconds based on published data^14^ and expert opinion. Interpretation of the trial results is based on the 95% confidence interval (CI) for the difference that lies wholly to the left of the noninferiority margin. If there is no difference between groups in surgical time, then 58 patients (29 per group) would provide 90% power to assess the noninferiority at a one-sided 2.5% significance level,^23^ assuming a common standard deviation (SD) of 70.2 seconds.^14^ For secondary outcomes, we explored evidence of a difference, rather than noninferiority.

Statistical analyses were performed using SPSS software (version 24.0; SPSS, Inc.). The Kolmogorov–Smirnov test was used to evaluate data distribution normality. Continuous data were expressed as mean ± SD, and between-group statistical comparisons were made by independent sample *t*-test or the Mann–Whitney *U* test, depending on normal distribution. Categorical data were described in numbers and percentages, and a Pearson χ^2^ analysis or a Fisher exact test was performed appropriately. A paired-sample *t*-test or a Wilcoxon signed-rank test was carried out to compare the questionnaire score data.

To account for the intereye correlation of some patients, we performed a sensitivity analysis on the perioperative parameters by analyzing data with marginal linear regression models using generalized estimating equations (GEEs).^24^^,^^25^ An exchangeable correlation structure was used in the models.

Two-sided 95% CIs of the difference between groups were reported for each outcome measure. Two-sided *P* values less than 0.05 were considered statistically significant.

## Results

### Patient Characteristics

A total of 242 eyes of 201 patients were included in the present study. Overall, 117 eyes and 125 eyes were enrolled in the 3D and TM groups, with a mean ± SD age of 69.61 ± 12.32 and 69.40 ± 11.82 years, respectively (Fig. 1). Demographic data and baseline characteristics are shown in Table 1. There were no significant differences between the two groups regarding these parameters.

**Table 1.** Baseline Characteristics of 3D Heads-up and TM Cataract Surgery Groups

Parameter	3D Group	TM Group	*P* Value
No. of eyes (No. of patients)	117 (112)	125 (109)	
Age, y	69.61 ± 12.32	69.40 ± 11.82	0.894^a^
Gender (women)	71 (60.7)	79 (63.2)	0.687^b^
Eye (right)	55 (47.0)	47 (52.0)	0.438^b^
Cataract grade			0.202^b^
Grade 1	33 (28.2)	24 (19.2)	
Grade 2	67 (57.3)	82 (65.6)	
Grade 3	13 (11.1)	12 (9.6)	
Grade 4	4 (3.4)	7 (5.6)	
Anterior chamber depth, mm	2.54 ± 0.50	2.49 ± 0.55	0.485^a^

Data are presented as mean ± standard deviation or *n* (%) unless otherwise indicated.

a Independent sample *t*-test.

b χ^2^ test.

### Patient Assessment

The mean ± SD surgical time was 462.03 ± 80.36 seconds for the 3D group and 452.13 ± 76.63 seconds for the TM group. There was no statistically significant difference between the two groups in terms of surgical time (*P* = 0.365, Table 2). In addition, the 95% CI for the difference (–9.98 to 29.78) did not include our noninferiority margin of 60 seconds. There were no observed differences in US power, APT, and EPT between the two groups (all *P* > 0.05, Table 2).

**Table 2.** Comparison of Intraoperative Parameters Between 3D Heads-up and TM Cataract Surgery Groups

Parameter	3D Group (*n* = 117)	TM Group (*n* = 125)	Difference (95% CI)	*P* Value
Surgical time, s	462.03 ± 80.36	452.13 ± 76.63	9.90 (–9.98 to 29.78)	0.365^a^
US power, %	14.86 ± 4.92	14.04 ± 4.89	0.82 (–0.45 to 2.08)	0.203^b^
APT, s	30.85 ± 17.50	28.82 ± 15.61	2.04 (–2.23 to 6.30)	0.382^a^
EPT, s	4.76 ± 4.11	4.29 ± 4.16	0.46 (–0.60 to 1.53)	0.220^a^

Data are presented as mean ± SD.

a Mann–Whitney *U* test.

b Independent sample *t*-test.

One-week follow-up data were available for 220 eyes (90.9%, *n* = 109 for the 3D group and *n* = 111 for the TM group). Table 3 displays the preoperative and postoperative results of UDVA, IOP, ECD, ECL, and CCT by group. Baseline parameters were comparable between the two groups (all *P* > 0.05). Improvement of visual acuity occurred postoperatively, regardless of surgical approach, while no significant differences were observed for the mean UDVA between the two groups (all *P* > 0.05). An increase in the IOP of the operated eyes was detected at 1 day postoperation, which decreased to preoperative values at 1 week in both groups. There was no statistically significant difference of IOP between the two groups throughout the follow-up (*P* = 0.619 at 1 day, *P* = 0.701 at 1 week). In both groups, there was a progressive decrease in ECD measurements postoperatively, and the values were not different significantly between the groups (*P* = 0.730 at 1 day, *P* = 0.171 at 1 week). The ECL was calculated at different time points during the follow-up; the *P* values for the between-group differences were 0.363 and 0.407 at 1 day and 1 week, respectively. CCT values increased in both groups after surgery compared with baseline and peaked at 1 day, yet the result showed no significant difference between the groups (*P* = 0.838 at 1 day, *P* = 0.882 at 1 week).

**Table 3.** Comparison of Perioperative Parameters Between 3D Heads-up and TM Cataract Surgery Groups

Parameter	3D Group	TM Group	Difference (95% CI)	*P* Value
Visual acuity (logMAR)				
Preoperative UDVA	0.77 ± 0.43	0.71 ± 0.39	0.06 (–0.05 to 0.16)	0.381^a^
1-day postoperative UDVA	0.26 ± 0.22	0.25 ± 0.26	0.01 (–0.05 to 0.07)	0.337^a^
1-week postoperative UDVA	0.30 ± 0.24	0.34 ± 0.26	–0.04 (–0.15 to 0.06)	0.461^a^
Intraocular pressure, mm Hg				
Preoperative	15.25 ± 3.30	15.40 ± 3.25	–0.15 (–0.98 to 0.68)	0.717^b^
1 day postoperative	16.20 ± 3.94	16.53 ± 4.24	–0.34 (–1.37 to 0.70)	0.619^a^
1 week postoperative	14.88 ± 3.45	15.07 ± 3.72	–0.19 (–1.17 to 0.79)	0.701^b^
Endothelial cell density, cells/mm^2^				
Preoperative	2585.77 ± 329.44	2551.22 ± 366.84	34.55 (–53.96 to 123.05)	0.480^a^
1 day postoperative	2342.10 ± 451.79	2349.44 ± 439.50	–7.34 (–125.38 to 110.69)	0.730^a^
1 week postoperative	2240.69 ± 524.52	2182.28 ± 445.54	58.41 (–107.99 to 224.81)	0.171^a^
Endothelial cell loss, %				
ECL at 1 day	8.85 ± 17.61	7.16 ± 15.05	1.68 (–2.64 to 6.00)	0.363^a^
ECL at 1 week	13.36 ± 21.96	13.58 ± 19.07	–0.22 (–7.25 to 6.82)	0.407^a^
Central corneal thickness, µm				
Preoperative	537.27 ± 36.60	531.74 ± 32.13	5.53 (–3.18 to 14.24)	0.212^b^
1 day postoperative	576.17 ± 64.95	572.27 ± 47.18	3.90 (–11.00 to 18.80)	0.838^a^
1 week postoperative	545.38 ± 38.59	544.42 ± 36.90	0.96 (–11.77 to 13.68)	0.882^b^

Preoperative and 1-day postoperative group size is 117 eyes for the 3D group and 125 eyes for the TM group. One-week postoperative group size is 109 eyes for the 3D group and 111 eyes for the TM group. Data are presented as mean ± SD. LogMAR, logarithm of the minimum angle of resolution.

a Mann–Whitney *U* test.

b Independent sample *t*-test.

In the sensitivity analysis, there was no significant difference in any perioperative parameters between the groups (all *P* > 0.05) in marginal linear regression models using GEEs, which was consistent with the main analysis (Supplementary Table S1).

All surgical procedures were uneventful, and complications were rare within both groups. Intraoperative and postoperative complications are presented in Table 4. One case of floppy iris (0.9%) occurred in the 3D group while no intraoperative complications were observed in the TM group. The incidence of IOP spike 1 day after operation was 7.7% (*n* = 9) in the 3D group and 5.6% (*n* = 7) in the TM group. At 1 week postoperation, IOP spike (*n* = 1, 0.9% for the 3D group; *n* = 3, 2.7% for the TM group) and mild corneal edema (*n* = 1, 0.9% for the 3D group; *n* = 3, 2.7% for the TM group) were more commonly observed than corneal epithelial punctate defect (*n* = 1, 0.9% for the TM group). No significant differences in complications were noted between the groups (all *P* > 0.05).

**Table 4.** Comparison of Perioperative Complications Between 3D Heads-up and TM Cataract Surgery Groups

Complication	3D Group, *n* (%)	TM Group, *n* (%)	Difference (95% CI), %	*P* Value
Intraoperative				
Floppy iris	1 (0.9)	0 (0.0)	0.9 (–0.8 to 2.5)	0.483^a^
Postoperative				
IOP spike at 1 day	9 (7.7)	7 (5.6)	2.1 (–4.2 to 8.4)	0.513^b^
IOP spike at 1 week	1 (0.9)	3 (2.7)	–1.8 (–5.3 to 1.7)	0.622^a^
Mild corneal edema at 1 week	1 (0.9)	3 (2.7)	–1.8 (–5.3 to 1.7)	0.622^a^
Corneal epithelial punctate defect at 1 week	0 (0.0)	1 (0.9)	–0.9 (–2.7 to 0.9)	1.000^a^

Intraoperative and 1-day postoperative group size is 117 eyes for the 3D group and 125 eyes for the TM group. One-week postoperative group size is 109 eyes for the 3D group and 111 eyes for the TM group.

a Fisher exact test.

b χ^2^ test.

### Questionnaire Evaluation

All observers, including 26 medical interns and 39 medical students, were asked to complete the questionnaire for both the 3D surgery and TM surgery. Scores are presented in Table 5. The 3D group had a significantly higher rating of satisfaction than the TM group for each parameter both before and after subgrouping (all *P* < 0.001), with the best results obtained for depth of field and educational value.

**Table 5.** Comparison of Observer Questionnaire Scores Between 3D Heads-up and TM Cataract Surgery Groups

Parameter	3D Group, Mean ± SD	TM Group, Mean ± SD	Difference (95% CI)	*P* Value
All observers (*n* = 65)				
Depth of field	9.40 ± 0.81	6.51 ± 1.51	2.89 (2.49 to 3.29)	<0.001^a^
Visibility	9.23 ± 0.98	7.98 ± 1.44	3.00 (2.29 to 3.71)	<0.001^a^
Detail understanding	9.08 ± 0.89	7.46 ± 1.52	2.82 (2.33 to 3.31)	<0.001^a^
Knowledge retention	8.95 ± 0.94	7.89 ± 1.30	1.25 (0.87 to 1.62)	<0.001^a^
Educational value	9.42 ± 0.81	8.06 ± 1.25	1.08 (0.55 to 1.60)	<0.001^a^
Medical interns (*n* = 26)				
Depth of field	9.42 ± 0.70	6.42 ± 1.58	1.36 (0.83 to 1.89)	<0.001^b^
Visibility	9.35 ± 0.80	8.27 ± 1.25	1.62 (1.24 to 1.99)	<0.001^b^
Detail understanding	9.23 ± 0.76	7.50 ± 1.68	1.73 (1.06 to 2.40)	<0.001^a^
Knowledge retention	9.15 ± 0.83	7.85 ± 1.35	1.54 (1.08 to 2.00)	<0.001^a^
Educational value	9.50 ± 0.65	8.04 ± 1.40	1.06 (0.76 to 1.37)	<0.001^a^
Medical students (*n* = 39)				
Depth of field	9.38 ± 0.88	6.56 ± 1.48	1.31 (0.75 to 1.86)	<0.001^a^
Visibility	9.15 ± 1.09	7.79 ± 1.54	0.90 (0.53 to 1.26)	<0.001^a^
Detail understanding	8.97 ± 0.96	7.44 ± 1.43	1.35 (1.03 to 1.68)	<0.001^a^
Knowledge retention	8.82 ± 1.00	7.92 ± 1.29	1.46 (0.89 to 2.04)	<0.001^a^
Educational value	9.36 ± 0.90	8.08 ± 1.16	1.28 (0.88 to 1.69)	<0.001^a^

a Wilcoxon signed-rank test.

b Paired sample *t*-test.

## Discussion

To our knowledge, this is the first study to report the efficacy of heads-up cataract surgery. Regardless of anterior or posterior segment surgery, the present study enrolled more than 240 eyes, making it the largest prospective randomized trial to comprehensively compare the clinical outcomes of heads-up surgery and TM surgery to date. We found similar intraoperative and postoperative parameters as well as complication incidence between the two groups. Moreover, the questionnaire results revealed that heads-up surgery was more valuable in medical education.

An increasing number of studies, including three small prospective trials with approximately 50 eyes in total,^4^^,^^16^^,^^18^ have revealed 3D surgery to be as safe and effective as TM surgery for vitreoretinal diseases.^15^^,^^17^^,^^19^ For the anterior segment, only a few reports focused on the application of 3D systems in DMEK^9^^,^^10^ and cataract surgery.^11^^–^^14^ Weinstock et al.^14^ discussed their initial experience of using the 3D visualization system for cataract surgeries in retrospect and reported that the surgical time and complications were comparable between the two groups. Another trial comprising 20 eyes also evaluated the feasibility of 3D cataract surgery.^13^ However, the small sample size may not have been able to provide sufficient statistical power, which might limit the generalizability of the findings. Our result showed that operation time was not significantly different between the two types of surgery performed by a single surgeon while controlling for other factors, indicating the feasibility of new surgical manipulation.

There is well-established evidence that ultrasound energy consumption is positively related to ECL.^26^^–^^28^ In the present study, similar intraoperative US power, APT, and EPT were observed in the two groups, which was consistent with the results of postoperative ECD and ECL. As expected, elevations in postoperative IOP and CCT were found in both the 3D and TM groups but were not of statistical significance. These results indicate that 3D surgery may not increase the inflammatory response and corneal edema over what is induced by TM surgery. Moreover, postoperative visual outcomes were improved and statistically equal between the two groups at both 1-day and 1-week follow-up.

All results mentioned above indicated that 3D surgery was not time-consuming and had comparable visual improvement and recovery efficacy to TM surgery. In addition, in line with a previous study,^14^ both groups displayed a similar and low incidence of complications, further demonstrating the safety of heads-up cataract surgery.

Heads-up technology may improve the teaching and learning of intraoperative procedures. In some cases, only surgeons could understand surgical details, but they could not communicate it to medical students by words or two-dimensional image. In 3D surgery, observers can see exactly the same high-quality stereoscopic surgical experience as a surgeon by wearing 3D glasses, helping them to observe more details and improve their understanding and knowledge retention.^6^ Therefore, in our results, medical interns and medical students’ satisfaction scores for 3D surgery were significantly better than for TM surgery, especially in terms of depth of field and educational value. These results were consistent with those of recent studies, which found that 3D technology was more comfortable for both beginner surgeons and observers regarding vitreoretinal diseases.^4^^,^^7^ Eckardt et al.^29^ reported that heads-up surgery was particularly suitable for surgical training in a situation where a teacher used cellular phones to instruct a trainee to perform surgeries. In that case, the teacher led the trainee through each individual step of the surgery, commenting frequently and in great detail. The trainee perceived this method as being more effective than receiving short, direct comments. An additional potential advantage of 3D systems with heads-up display in the teaching field is that the surgical assistant and students are able to view the steps of surgery while maintaining adequate interpersonal distance, which has become important since the coronavirus disease 2019 pandemic began.^30^

Heads-up surgery has several advantages over TM surgery. In addition to the educational value mentioned above, the most beneficial advantage is its ergonomic design, which enables a more physiologically comfortable and stable body posture to relieve fatigue and musculoskeletal stress for surgeons, thereby extending their careers.^2^^–^^5^ This view is in accordance with some surgeon-oriented questionnaire studies.^4^^,^^5^^,^^7^^,^^11^^,^^31^

With the development of 5G data transmission and virtual reality technology, ophthalmologists are expected to experience remote cataract surgery broadcasting easily in the near future, which will benefit learning and communication using surgical technology. Heads-up surgery may overcome the visualization limitations of standard microsurgery with increased magnification, extended depth of field, and improved depth resolution, enabling surgeons to distinguish intraocular tissue structure better.^2^ In addition, 3D visualization system can decrease illumination to reduce the risk of phototoxicity.^5^^,^^32^^,^^33^ With the screen being approximately 2.2 m away from the surgeon, as shown in Figure 2, a wider field of view is available to the surgeon compared with looking down through the eyepiece. The surgeon can magnify the image to a high magnification without experiencing any discomfort or eyestrain under the same microscope magnification, thereby relieving eye fatigue.^2^ In addition, surgeons can more conveniently receive instruments from technicians by observing from the corners of their eyes, which improves operational efficiency.

The current technology still has room for improvement. The learning curve is the primary issue with implementing a new technology. The setup of the 3D screen, best surgical posture, and magnification vary by surgeon and require gradual adjustment and adaption. However, the learning curve seems to be short, since it has been reported that just a few surgical practices are enough to make the surgeon feel familiar and comfortable with 3D surgery.^2^^,^^7^^,^^31^

Some 3D visualization systems only retain the heads-up path and cover the microscopic eyepiece. This design may cause issues for beginning heads-up surgeons if they encounter complex or unexpected situations. For example, eye socket hydrops occurs more often in patients with narrow palpebral fissure, resulting in increased reflected light. In this circumstance, microsurgical procedures conducted with a viewing screen can be difficult and unsafe. Therefore, both a heads-up path and microscope eyepiece should be available and switched between as needed in surgery.

In terms of the visualization system, system delay between the steps of surgery and the video projected on the screen is difficult to avoid and can lead to deviations in sophisticated operation. The lag may be more evident in anterior segment surgeries due to the higher instrumental speed during surgical manipulations. However, in the present study, we did not find the duration to be extended significantly in 3D surgery performed by an experienced surgeon. The surgeon also did not report any delay between the procedure and the screen display throughout the cataract surgery.

The present study had several limitations. First, the follow-up period was only 1 week. For patients who received uncomplicated small-incision surgery, they usually recovered well a short term after surgery. We set the follow-up period as 1 week because the efficacy and safety outcomes were supposed to generally get stabilized at 1 week.^20^ Second, to maintain better surgical homogeneity, only one experienced surgeon used both approaches to perform surgery. In the future, multicenter randomized controlled trials with multiple surgeons and a larger patient sample size should be conducted to confirm our observations and evaluations. Third, in our questionnaire study, the observers rated their satisfaction with knowledge retention and educational value by completing the 10-point scale questionnaire rather than the posttest assessment. However, none of the observers had the qualification to perform a cataract surgery. As such, the aim of our questionnaire study is to preliminarily evaluate the perception of the observers regarding the learning-teaching methodology. Follow-up studies are warranted to further investigate the teaching value of the heads-up cataract surgery by posttest assessment. Fourth, even with a sample size of more than 100 eyes per group, we cannot comment on the incidence of the rare but severe complications that do occur with cataract surgery. Fifth, the statistical comparisons in our study were not adjusted for multiple comparisons. Sixth, the learning curve of surgeons is worthy of further investigation.

## Conclusions

In conclusion, the current study prospectively demonstrates that heads-up cataract surgery has comparable efficiency, efficacy, and safety to TM surgery in femtosecond laser-assisted cataract surgery when an experienced surgeon is performing both procedures. Moreover, heads-up surgery shows a significant advantage in terms of teaching and learning intraoperative surgical procedures. It is hoped that with continuous refinement, the 3D visualization system can shorten system delay and improve resolution to serve patients, surgeons, and the development of ophthalmic surgery better.

## Supplementary Material

Supplement 1
