# Supplementary material for: Three-Dimensional Heads-up Cataract Surgery Using Femtosecond Laser: Efficiency, Efficacy, Safety, and Medical Education—A Randomized Clinical Trial
Source: Transl Vis Sci Technol. 2021 Aug 3;10(9):4. doi: 10.1167/tvst.10.9.4 (PMC8340661; doi:10.1167/tvst.10.9.4)
Supplement: Supplement 1 [file tvst-10-9-4_s001.pdf]

Supplementary Table S1. Surgical technique (3D vs. TM) affecting the changes in perioperative parameters were analyzed with marginal linear regression models using the GEE.

Parameters	Regression coefficient	95% Confidence Intervals	*p* value
Surgical time (second)	0.02	(-0.02 to 0.06)	0.37
US power (%)	0.05	(-0.04 to 0.14)	0.28
APT (second)	0.05	(-0.08 to 0.18)	0.47
EPT (second)	0.05	(-0.16 to 0.26)	0.65
Visual acuity (logMAR)			
preoperative UDVA	0.07	(-0.06 to 0.21)	0.30
1 day postoperative UDVA	0.06	(-0.16 to 0.27)	0.62
1 week postoperative UDVA	-0.13	(-0.43 to 0.17)	0.39
Intraocular pressure (mmHg)			
preoperative	8.0×10^{-4}	(-0.05 to 0.05)	0.97
1 day postoperative	-0.04	(-0.11 to 0.03)	0.26
1 week postoperative	0.03	(-0.06 to 0.11)	0.50
Endothelial cell density (cells/mm2)			
preoperative	0.02	(-0.02 to 0.05)	0.33
1 day postoperative	4.4×10^{-3}	(-0.04 to 0.05)	0.86
1 week postoperative	0.04	(-0.04 to 0.11)	0.35
Endothelial cell loss (%)			
ECL at 1 day	0.02	(-0.29 to 0.33)	0.91
ECL at 1 week	0.06	(-0.27 to 0.39)	0.74
Central corneal thickness (μm)			
preoperative	0.01	(-4.0×10^{-5} to 0.02)	0.05
1 day postoperative	0.01	(-0.01 to 0.03)	0.56
1 week postoperative	0.01	(-4.1×10^{-3} to 0.02)	0.21

Note. 3D = 3D heads-up cataract surgery; TM = traditional microscopic cataract surgery; GEE = generalized estimating equations; US = ultrasound; APT = absolute phacoemulsification time; EPT = effective phacoemulsification time; LogMAR = logarithm of the minimum angle of resolution; UDVA = uncorrected distance visual acuity; ECL = endothelial cell loss. Preoperative, intraoperative and 1 day postoperative group size is 117 eyes for 3D group and 125 eyes for TM group. One week postoperative group size is 109 eyes for 3D group and 111 eyes for TM group. TM is used as the reference category in the GEE approach.
